# Supplementary material for: [2+2] Cyclo-Addition Reactions for Efficient Polymerization on a HOPG Surface at Ambient Conditions
Source: Nanomaterials (Basel). 2022 Apr 13;12(8):1334. doi: 10.3390/nano12081334 (PMC9031210; doi:10.3390/nano12081334)
Supplement: Supplementary file 1 [file nanomaterials-12-01334-s001.zip › nanomaterials-1642676-suppl proof back/SI/SI_164676.pdf]

nanomaterials

MDPI

Article

[2+2] Cyclo-addition Reactions for Efficient Polymerisation on a HOPG Surface at Ambient Conditions

Lihao Guan [1], Frank Palmino [2] Jean-Christophe Lacroix [1], Frédéric Chérioux [2,*] and Xiaonan Sun [1,*]

[1] Université de Paris, ITODYS, CNRS, 15 rue Jean Antoine de Baïf, 75013 Paris, France
[2] Université de Franche-Comté, FEMTO-ST, CNRS, UFC, 15B avenue des Montboucons 25030 Besancon cedex, France.
* Correspondence: FC: frederic.cherioux@femto-st.fr ; XS: sun.xiaonan@univ-paris-diderot.fr

(a)

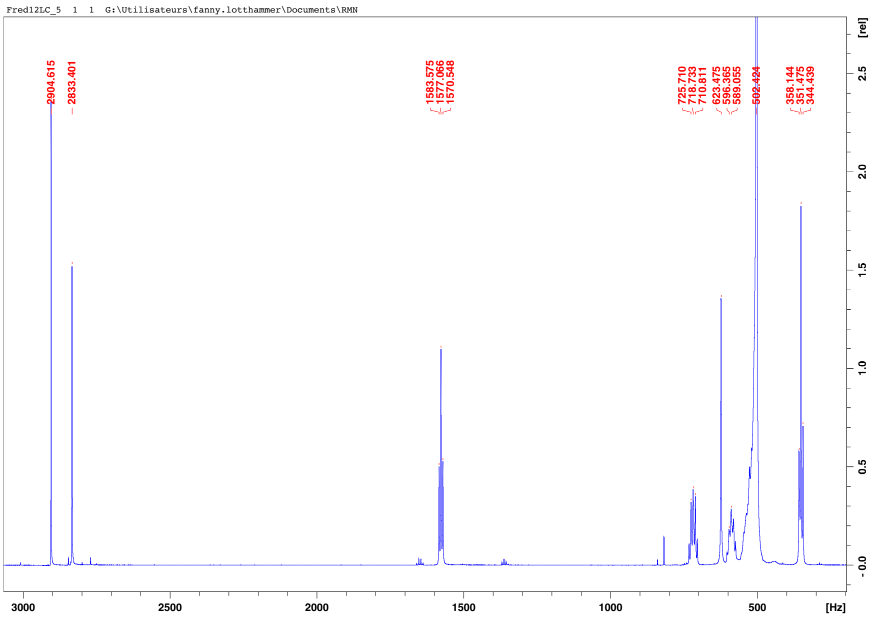

(b)

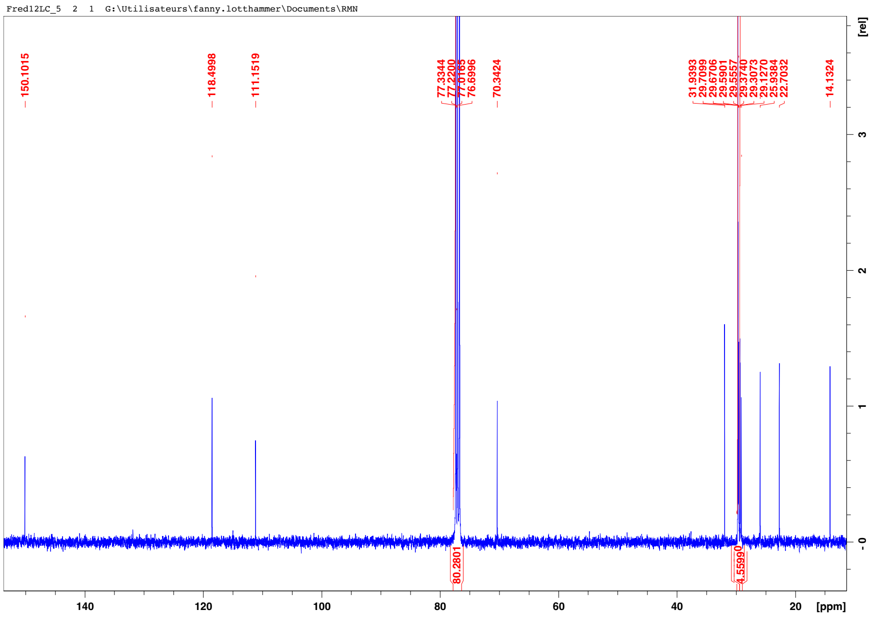

Figure S1. (a) ^1H NMR and (b) ^{13}C spectra of 1,4-dibromo-2,5-bis(octadecyloxy)benzene ($CDCl_3$).

(a)

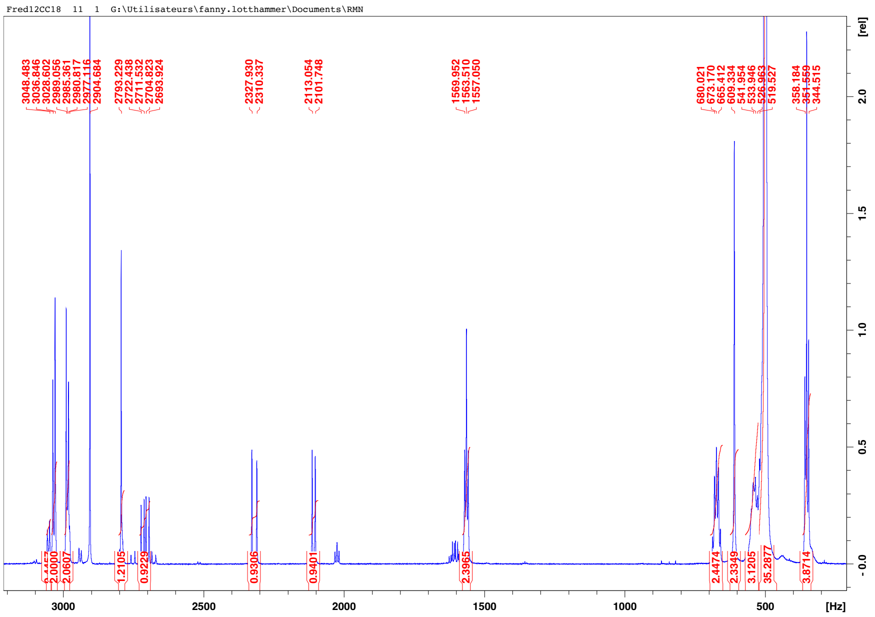

(b)

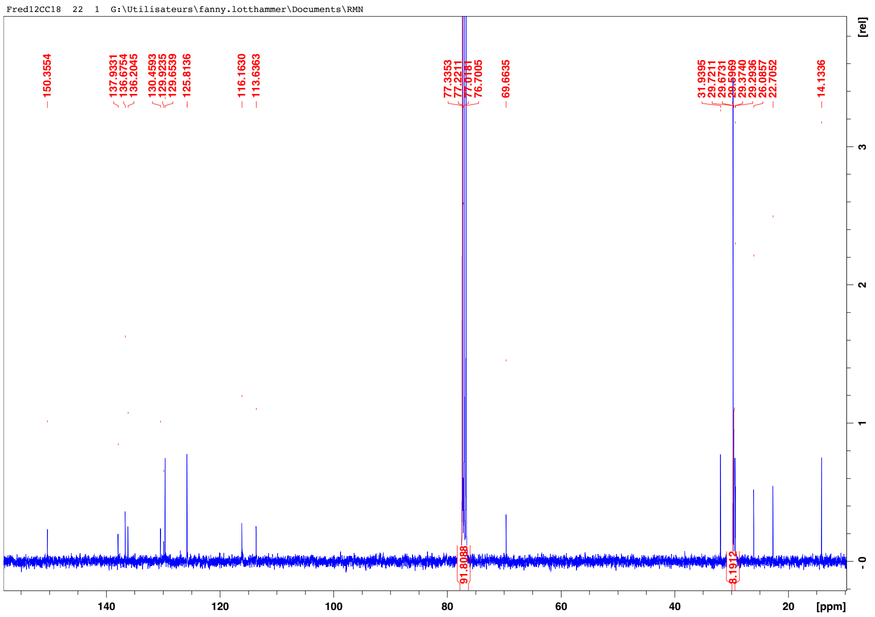

Figure S2. (a) ^{1}H NMR and (b) ^{13}C spectra of 1,4-bis(4′-vinylphenyl)-2,5-bis(octadecyloxy)benzene ($CDCl_3$).

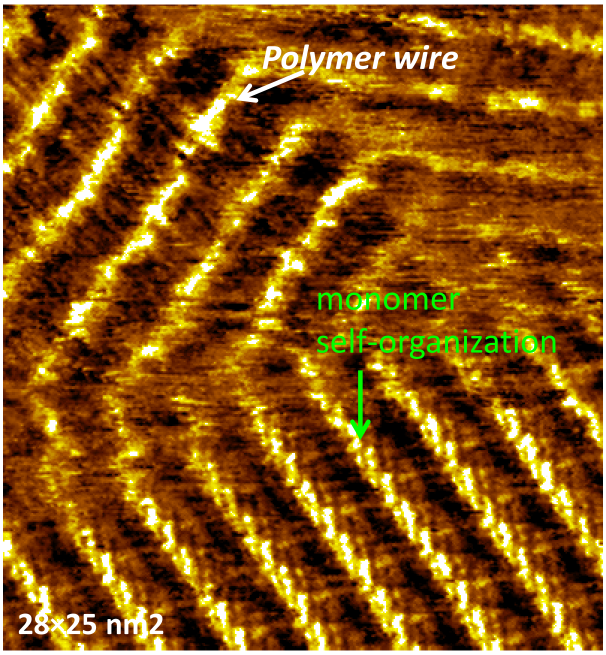

Figure S3. STM image (Vs = -0.5V, It= pA, 28 nm x 25 nm) of vinyl-OC18 photo-polymerized (upper part) and unpolymerized (lower part) networks. 31

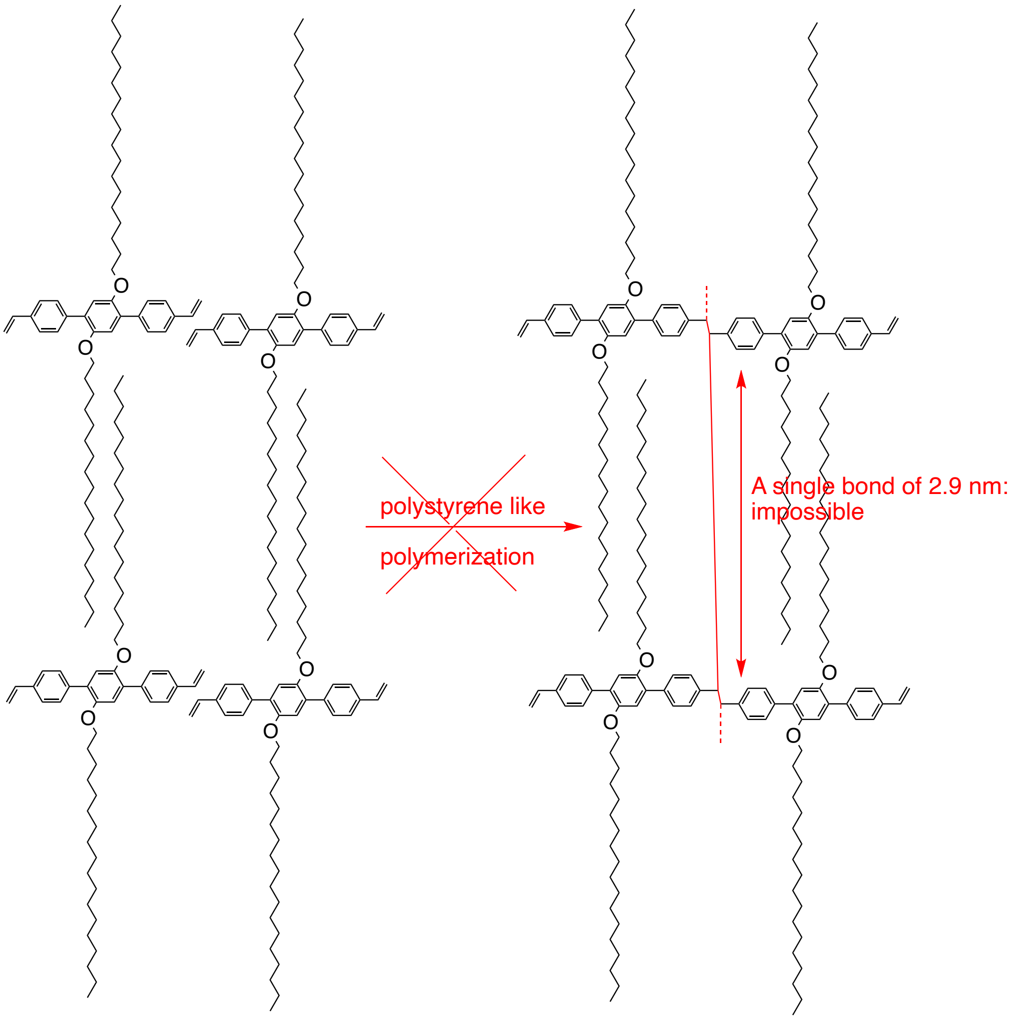

Figure S4. Scheme of hypothetic polymerization of C=C bonds between adjacent vinyl-OC18 molecules as usually observed in the case of styryl-derivatives. The lateral alkyl chains are too long to allow the formation of C-C bond between molecules (highlighted in red).

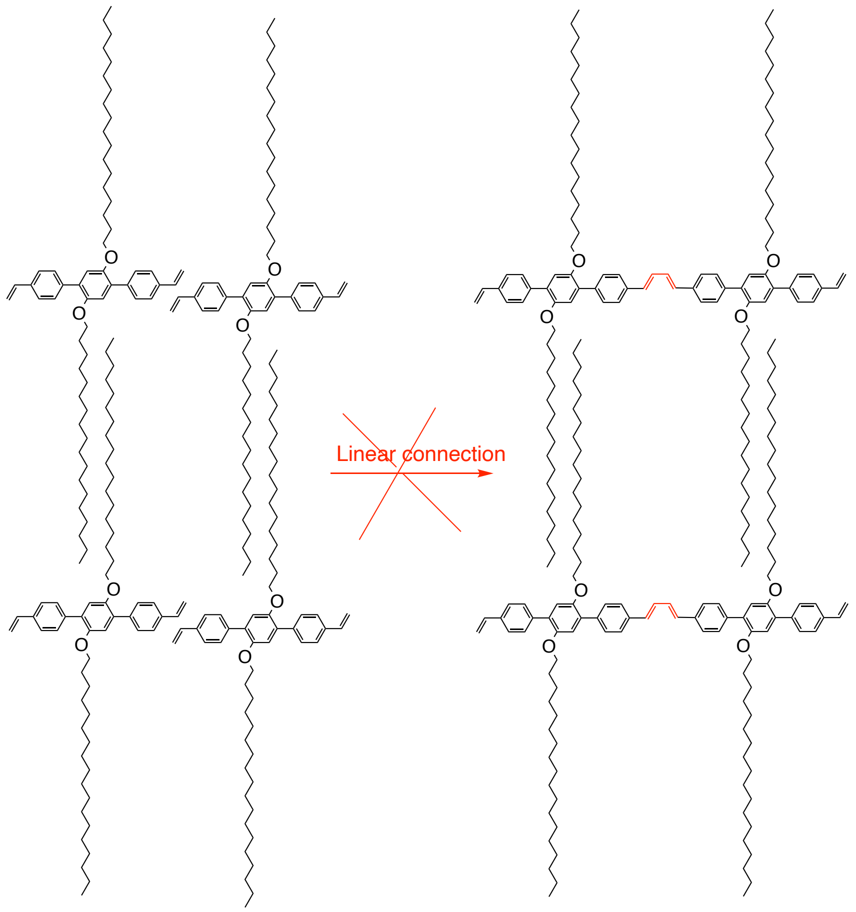

Figure S5. Scheme of hypothetic polymerization of C=C bonds between consecutive vinyl-OC18 molecules (new bonds are highlighted in red). This type of reaction requires coinage metal surface, high temperature and UHV conditions.
